# EXECUTING AN ENVIRONMENTAL SCAN OF UNIVERSITY AGE-FRIENDLINESS: FINDINGS FROM A MIXED METHODS STUDY

**DOI:** 10.1093/geroni/igz038.3318

**Published:** 2019-11-08

**Authors:** Andrea G Zakrajsek, Sarah Masinda, Amanda Simon, Cassandra Barragan

**Affiliations:** Eastern Michigan University, Ypsilanti, Michigan, United States

## Abstract

Internationally, universities are recognizing the importance of understanding and enhancing age as a component diversity and inclusion efforts through the Age-Friendly University (AFU) initiative. This session will describe an environmental scan of “age-friendliness” that one AFU university designed. The overall aims of the project include: (1) exploring how stakeholders understand age-friendliness, (2) identifying current efforts and opportunities that exist within the university, and (3) gathering data that describes the perception of barriers that older learners encounter at the university. This presentation will be used to discuss a mixed-methods study that included interviews and a survey of performance and importance ratings of the international AFU principles. Twenty-eight participants were purposefully recruited from divisions across a campus of a regional university to participate in in-depth interview data collection with the research team. Qualitative thematic findings that emerged through a constant comparative method of analysis of interview transcripts include: Existence of Age-Inclusivity Barriers and Opportunities for Change, Need for Intentionality in Age-Friendly Efforts, and Importance of Connections. Furthermore, AFU principle performance and importance ratings were descriptively analyzed in order to prioritize university efforts to enhance inclusion initiatives related to age.

